# Association of the insulinemic potential of diet and lifestyle with risk of diabetes incident in Tehranian adults: a population based cohort study

**DOI:** 10.1186/s12937-021-00697-2

**Published:** 2021-04-23

**Authors:** Hossein Farhadnejad, Ebrahim Mokhtari, Farshad Teymoori, Mohammad Hassan Sohouli, Nazanin Moslehi, Parvin Mirmiran, Fereidoun Azizi

**Affiliations:** 1 Nutrition and Endocrine Research Center, Research Institute for Endocrine Sciences, Shahid Beheshti University of Medical Sciences, Tehran, Iran; 2 Department of Nutrition, School of Public Health, Iran University of Medical Sciences, Tehran, Iran; 3 Endocrine Research Center, Research Institute for Endocrine Sciences, Shahid Beheshti University of Medical Sciences, Tehran, Iran

**Keywords:** Empirical indices, Dietary patterns, Lifestyle indices, Hyperinsulinemia, Insulin resistance, Type 2 diabetes

## Abstract

**Background:**

We aimed to assess the associations between insulinemic potential of diet and lifestyle and the risk of diabetes incident, using four empirical indices including the empirical dietary index for hyperinsulinemia (EDIH), the empirical dietary index for insulin resistance (EDIR), empirical lifestyle index for hyperinsulinemia (ELIH), and empirical lifestyle index for insulin resistance (ELIR).

**Methods:**

A total of 3734 individuals, aged ≥ 20 years old, who were free of diabetes at baseline (2008–2011), were followed for 6.2 years (2015–2018) to ascertain incident diabetes. The food frequency questionnaire was used to collect dietary intakes at baseline. Odds ratio (OR) of diabetes were calculated across quartiles of EDIH, EDIR, ELIH, and ELIR using logistic regression, which controlled for confounding factors.

**Results:**

The mean ± SD age and BMI of individuals (45.1 % male) were 40.9 ± 12.0 years and 27.1 ± 4.1 kg/m^2^, respectively. At the end of follow-up, 253 (6.8 %) diabetes cases were identified. In the multivariable-adjusted model, individuals in the highest quartile of EDIR (1.58;95 %CI:1.03–2.44, *P* for trend = 0.025), ELIH (1.89;95 %CI:1.20–2.97, *P* for trend = 0.004), and ELIR (1.74; 95 %CI:1.11–2.72, *P* for trend = 0.031) had increased the risk of diabetes. However, no significant associations were found between the score of EDIH and diabetes incident.

**Conclusions:**

Higher adherence to EDIR, ELIH, and ELIR scores were associated with increased risk of diabetes, while no significant association was found between EDIH score and diabetes incident.

## Background

Diabetes is a common chronic disease that is rapidly increasing globally [1]. It is anticipated that by 2035 the number of diabetes worldwide will reach 592 million. More than 80 % of diabetes cases live in low- and middle-income countries that resources for prevention, control, and management of diabetes are limited [2]. It is estimated that the incidence rate of type 2 diabetes in Iran is 36.3 per 1000 person-years, with more than 800,000 new cases per year [3]. Diabetes is a multifactorial disease that develops as a result of complex interactions between genes and environmental factors such as a sedentary lifestyle, emotional stress, socioeconomic status, unhealthy dietary intakes, and metabolic abnormalities [1, 4].

Insulin resistance (IR) and hyperinsulinemia as metabolic disorders play important roles in the onset of diabetes and its progression [5, 6]. In fact, impaired insulin secretion along with inadequate peripheral tissue response to insulin is the main and direct cause of diabetes through dysregulating of energy and glucose metabolism. Moreover, C-peptide concentrations are considered as a valid marker of hyperinsulinemia which can predict the incidence of diabetes [7, 8]. The collection of environmental factors in the form of lifestyle, including adiposity, physical inactivity, smoking, and alcohol consumption are suggestive factors that may be strongly linked to IR and hyperinsulinemia in adults. Also, the dietary insulinemic potential may be associated with hyperinsulinemia and IR independent of total energy and macronutrient intakes.Therefore, these factors as major determinants of IR and hyperinsulinemia may increase consequently the risk of chronic diseases such as diabetes [1, 9, 10].

Previously, some empirical indices were developed to assess the insulinemic potential of diet and lifestyle, termed the empirical dietary index for hyperinsulinemia (EDIH), empirical lifestyle index for hyperinsulinemia (ELIH), the empirical dietary index for IR (EDIR), and the empirical lifestyle index for IR (ELIR) [11]. Also, recently, several studies have indicated the potential link between above mentioned empirically derived dietary and lifestyle indices and risk of chronic diseases such as digestive system cancer risk [12], colorectal cancer [13], and also overweight or obesity [14]. To the best of our knowledge, evidence on the association between the insulinemic potential of diet and lifestyle score and risk of diabetes is limited to a Jin et al. study, indicating a positive link between EDIH and increased risk of diabetes [15], however, the association of EDIR, ELIH, and ELIR with the risk of hyperinsulinemia, IR, and incidence of diabetes have not yet been investigated.

Given the lack of convincing evidence regarding the association of the insulinemic potential of diet and lifestyle with diabetes incident, we aimed to investigate the longitudinal assoations between insulinemic potential of diet or lifestyle and risk of diabetes in a cohort of Iranian adults.

## Methods

### Study population

The current study was performed within the framework of the Tehran Lipid and Glucose Study (TLGS), a population-based prospective study that was conducted to determine the risk factors for chronic diseases among a representative urban population of Tehran, including 15,005 participants, aged ≥ 3 years [16]. The first survey of TLGS (a cross-sectional survey) is initiated in March 1999 and data collection, conducted prospectively at 3 years intervals, is ongoing; the details of the TLGS have been reported previously [16].

**Fig. 1:**
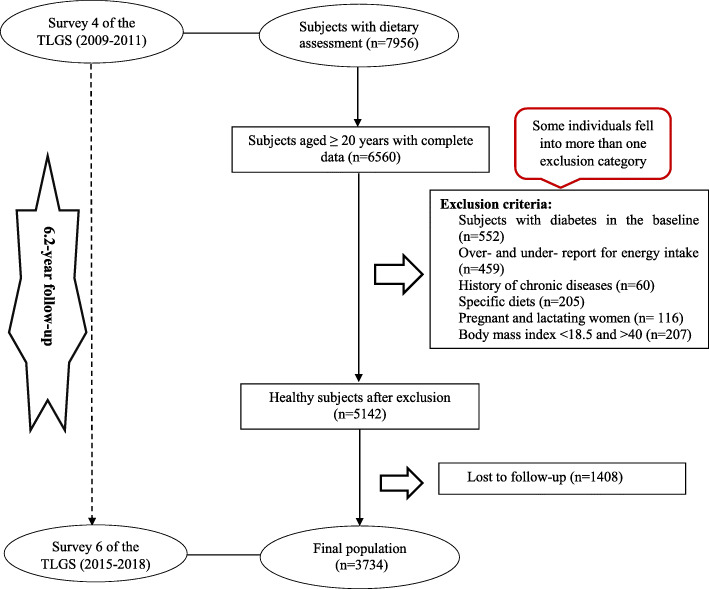
Flow chart of the Tehran Lipid and Glucose Study (TLGS) participants

In the fourth examination of the TLGS (2009–2011), from 12,823 participants, 7956 randomly selected, agreed to complete the dietary assessment. For the current study, a total of 6560 individuals, aged ≥ 20 years old, with complete data in the fourth examination of TLGS, as a baseline examination, were enrolled. Subjects with underreporting or over-reporting dietary intakes (less than 800 kcal/d or more than 4500 kcal/d, respectively), (*n* = 459) or on hyperglycemic diets (*n* = 205); those with a history of myocardial infarction, cerebral vascular accident, and cancers (*n* = 60); those with diabetes (*n* = 552); those with non-normal body mass index (lower than 18.5 and upper than 40), (*n* = 207), and lactating and pregnant women (*n* = 116) were excluded. Some individuals fell into more than one exclusion category. Finally, 5142 participants were followed-up until the sixth phase of TLGS (2015-18), over a mean period of 6.2 years. After excluding the participants who were missed to follow up (*n* = 1408), final analyses was conducted on 3734 adult subjects (Fig. 1).

### Physical activity assessment

The physical activity levels of participants were assessed using the modifiable activity, which has previously been modified and validated among the Iranians population [17]; individuals were asked to report and identify the frequency and time spent on activities of light, moderate, hard, and very hard intensity, during the past 12 months, according to a list of common activities of daily life; physical activity levels were expressed as metabolic equivalent hours per week(Met.h.wk).

### Clinical and biological measurements

A pretested questionnaire was used by trained interviewers to collect the information of participants on age, sex, medical history, medication use, and smoking habits. The participant’s weight was measured and recorded in light clothing, without shoes or socks, using a digital scale (model 707, Seca, Hamburg, Germany) with an accuracy of up to 100. Height was measured in a standing position without shoes, using a stadiometer to the nearest 0.1 cm (model 208 Portable Body Meter Measuring Device; Seca). Body mass index (BMI) was computed as weight (kg) divided by height (m^2^). Waist circumference (WC) was measured to the nearest 0.1 cm using an un-stretched tape meter, at the level of the umbilicus, over light clothing, without any pressure on the body surface.

Blood samples were taken and transferred into vacutainer tubes between 7:00 and 9:00 a.m, after a 12–14-h overnight fast, while subjects were in sitting position. Blood samples were centrifuged within 30 to 45 min of collection. All biochemical analyses were performed using a Selectra 2 auto-analyzer at the TLGS research laboratory, on the day of blood collection. Fasting blood sugar (FBS) was measured using an enzymatic colorimetric method with glucose oxidase. Inter- and intra-assay CVs were both 2.2 % for FBS [16]. The 2-h oral glucose tolerance test was performed using a 82.5 g of glucose monohydrate solution (equivalent to 75 g anhydrous glucose), which administered orally to the all individuals aged > 20 years, except diabetic patients on anti-diabetic drug therapy based on prescription of endocrinologist. Triglyceride (TGs) levels were measured using the enzymatic colorimetric method with glycerol phosphate oxidase. Inter- and intra-assay CVs for TGs were 0.6 and 1.6 %, respectively. Serum high-density lipoprotein-cholesterol (HDL-C) was measured after precipitation of the apolipoprotein B-containing lipoproteins with phosphotungstic acid. Enzymatic colorimetric tests were used to assay total cholesterol (TC) with cholesterol esterase and cholesterol oxidase. Inter- and intra-assay CVs for both TC and HDL-C were 0.5 and 2 %, respectively. Analyses were performed using commercial kits (Pars Azmoon Inc., Tehran, Iran).

### Dietary intake assessment

Dietary intakes of participants over the previous year were assessed using a valid and reliable semi-quantitative food frequency questionnaire (FFQ) at baseline [18]. The reliability and validity of the FFQ have been previously reported. Consumption frequency for each food item during the previous year on a daily, weekly, or monthly basis was collected during a face-to-face interview by trained and experienced dieticians. Portion sizes of consumed foods, reported in household measures were then converted into grams. Using the United States Department of Agriculture (USDA) food composition table (FCT), energy and nutrient contents were computed. The Iranian FCT was used for local food items that were not available in USDA FCT.

### Calculation of indices

Dietary data derived from FFQ were used to calculate insulinemic scores. Calculating the EDIH and ELIH has been explained elsewhere ) [11]. EDIH score; calculated with 15 instead of 18 food groups including processed meat (sausage), red meat (beef, or lamb), fish (canned tuna, or fish), margarine, poultry (chicken with or without skin), French fries, high-energy beverages (cola with sugar, carbonated beverages with sugar, fruit punch drinks), tomatoes, low-fat dairy products (skimmed or low-fat milk and yogurt), and eggs (positive association) and also, coffee, green leafy vegetables (cabbage, spinach, or lettuce), whole fruits, and high-fat dairy products (whole milk, cream, cream cheese, and other cheese) )inverse association(.

ELIH score; calculated with 11 instead of 14 dietary and lifestyle factor including BMI, margarine, butter, red meat, and fruit juice (apple juice, cantaloupe juice, orange juice, or other fruit juice) with positive association and coffee, whole fruit, physical activity, high-fat dairy products, snacks, and salad dressing with the inverse association.

Since consumption of alcoholic drinks such as wine and liquor is unusual in the Iranian population due to religious considerations and wasn’t reported in the TLGS study, we don’t include them in the calculation of indices. As we had not any food items as low energy beverages and cream soup in our FFQ we excluded them in the calculation.

For weighting, the daily intakes of each food group (serving size) and lifestyle factors values multiplied by specific proposed regression coefficients. Finally, to calculate total scores, all values of weighted food group and lifestyle factors were summed and then divided by 1000 to decline the magnitude of the scores which ease the interpretation of the results.

EDIR score; calculated with 13 instead of 18 food Items including margarine, red meat, refined grains, processed meat, tomatoes, other vegetables, fish, fruit juice (positive association), and coffee, green leafy vegetables, high-fat dairy products, dark yellow vegetables, nuts (inverse association).

ELIR score; calculated with 14 instead of 17 dietary and lifestyle factor including BMI, refined grains, red meat, margarine, tomatoes, other vegetables, potatoes, fruit juice, processed meat, tea with positive association and coffee, green leafy vegetables, high-fat dairy products, physical activity with the inverse association.

Weighting and calculation of EDIR and ELIR were conducted similar to which was mentioned above for EDIH and ELIH.

### Definitions of terms

Type 2 diabetes was defined based on the criteria of the American Diabetes Association (ADA) as FPG ≥ 126 mg/dl or 2-h post 75-gram glucose load ≥ 200 mg/dl or being on anti-diabetic medication [19].

### Statistical analysis

Data were analyzed using the Statistical Package for Social Sciences (version 20.0; SPSS Inc, Chicago IL). The normality of variables was checked using histogram charts and Kolmogorov–Smirnov tests. Participants were classified into quartiles based on scores of EDIH, EDIR, ELIH, and ELIR. Baseline characteristics of participants in quartiles of EDIH and EDIR were expressed as mean ± SD or median (interquartile range (IQR)) for continuos variables, and number (percentage) for categorical variables. To test the trend of qualitative and quantitative variables across quartiles EDIH and EDIR, Chi-square and linear regression were used, respectively. The associations of EDIH, EDIR, ELIH, and ELIR with diabetes incident were assessed using multivariable logistic regression models. The odds ratios (ORs) and 95 % confidence interval (CI) was reported based on three adjusted models for confounder variables including model 1 (adjusted for age and sex), model 2 [additional adjustment for waist circumference (for EDIH and EDIR), waist adjusted BMI (for ELIH and ELIR), smoking, physical activity (for EDIH and EDIR), education level, and energy intake], and model 3 (additional adjustment for baseline values of fasting blood sugar, and TAG: HDL-cholesterol). *P*-values < 0.05 were considered to be statistically significant.

## Results

The mean ± SD for the age and BMI of the participants (45.1 % male) were 40.9 ± 12.0 years and 27.1 ± 4.1 kg/m^2^, respectively. Also, the median (IQR) of EDIH, ELIH, EDIR, and ELIR was 0.10 (0.01–0.23), 1.26 (1.11–1.43), 0.60 (0.40–0.84), and 3.44 (2.67–4.79), respectively. During an average of 6.2 years of follow-up, 253 (6.8 %) new cases of diabetes were identified.

The Baseline characteristics and dietary intake of participants according to the EDIH score are shown in Table 1. Participants in the highest EDIH score quartile were more likely to be male, smoker, and having higher academic education, they were also younger, have higher physical activity, andlower BMI, WC, and FBS concentration, compared to those in the lowest one (*P* < 0.05). Also, participants in the highest quartile of EDIH score had higher intakes of low-fat dairy, vegetables, refined grain, red and processed meat, but lower intakes of fruits and high-fat dairy. Dietary intakes of total energy and total fat were significantly increased across EDIH score quartiles (*P* < 0.001), whereas the intake of carbohydrates was decreased (*P* < 0.001). There were no significant differences in any other demographic, anthropometric, biochemical, and nutritional measures across quartiles of the EDIH score.

**Table 1:** Baseline characteristics of participants according to quartiles (Q) of the empirical dietary index for hyperinsulinemia

	Empirical dietary index for hyperinsulinemia				*P* for trend*
	Q1(*n*=933)	Q2(*n*=933)	Q3(*n*=933)	Q4(*n*=933)	
**Demographic and Anthropometric data**					
Age (years)	42.8(12.8)	42.1 (12.5)	40.4 (11.8)	38.8 (11.5)	<0.001
Male (%)	354 (37.9)	419 (44.9)	422 (45.2)	491 (52.6)	<0.001
Physical activity (MET-h/wk)	64.6 (30.8 – 95.9)	65.5 (32.9 – 93.4)	67.6 (38.5 – 102.6)	72.7 (39.8 -104.9)	<0.001
BMI (kg/m2)	27.6 ± 3.9	27.1 ± 4.2	27.2 ± 4.2	26.5 ± 4.0	<0.001
waist circumference (cm)	92.6 ± 10.9	92.0 ± 11.1	92.0 ± 11.0	91.1 ± 10.7	0.005
Current smokers (%)	77 (8.3)	87 (9.3)	106 (11.4)	134 (14.4)	<0.001
Academic education, N (%)	215 (23.0)	273 (29.3)	301 (32.3)	300 (32.2)	<0.001
**Biochemical data**					
FBS(mg/dl)	93.1 ± 8.4	93.0 ± 8.4	93.0 ± 8.1	92.3 ± 8.4	0.044
TGs(mg/dl)	139 ± 86	140 ± 85	136 ± 80	136 ± 78	0.299
HDL- Cholesterol (mg/dl)	47.8 ± 12.2	48.1 ± 11.4	47.5 ± 11.1	47.1 ± 11.2	0.078
TG:HDL ratio	3.23 ± 3.06	3.27 ± 2.68	3.20 ± 2.45	3.26 ± 2.63	0.635
**Insulin Scores**					
EDIH	-0.05 ± 0.08	0.06 ± 0.02	0.16 ± 0.03	0.44 ± 0.29	<0.001
ELIH	1.18 ± 0.22	1.24 ± 0.23	1.29 ± 0.23	1.40 ± 0.30	<0.001
EDIR	0.50 ± 0.35	0.61 ± 0.31	0.73 ± 0.35	0.83 ± 0.48	<0.001
ELIR	3.56 ± 1.87	3.88 ± 1.86	4.14 ± 1.84	4.43 ± 2.11	<0.001
**Nutrient Intake**					
Energy(Kcal/d)	2303 ± 750	2241 ± 703	2401 ± 685	2758 ± 719	<0.001
Carbohydrate(% of energy)	61.1 ± 7.5	59.9 ± 6.0	59.0 ± 12.5	55.8 ± 6.3	<0.001
Protein(% of energy)	14.5 ± 3.6	14.9 ± 4.0	15.6 ± 12.8	14.9 ± 3.2	0.299
Fat(% of energy)	28.8 ± 6.4	28.8 ± 5.9	30.4 ± 29.0	32.3 ± 6.2	<0.001
**Food groups**					
Low-fat dairy (serving/d)	1.81 ± 1.23	1.71 ± 1.09	1.86 ± 1.13	1.96 ± 1.26	<0.001
High-fat dairy (serving/d)	0.88 (0.22 – 1.41)	0.45 (0.16 – 1.04)	0.47 (0.16 – 0.99)	0.55 (0.22 – 1.09)	<0.001
Refined grain(serving/d)	4.16 ± 3.01	4.58 ± 2.97	4.91 ± 3.06	5.32 ± 3.36	<0.001
Red and processed meat(serving/d)	0.57 ± 0.41	0.69 ± 0.48	0.90 ± 0.61	1.18 ± 1.05	<0.001
Fruits(serving/d)	5.19 ± 4.70	3.41 ± 2.93	3.33 ± 2.83	3.47 ± 2.77	<0.001
Vegetables(serving/d)	3.24 ± 2.28	3.04 ± 1.79	3.49 ± 2.10	3.82 ± 2.70	<0.001

Data represented as mean ±SD, or median (IQR 25-75) for continuous variables and number and percent for categorical variables

*Chi-square and linear regression were used to test the trend of continuous and categorical variables across quartiles of the empirical dietary index for hyperinsulinemia (as the median value in each quartile), respectively

Also, the baseline characteristics of study participants according to the EDIR score are presented in Table 2. Participants in the highest EDIR score quartile were more likely to be male, younger, and have higher physical activity, academic education, TGs, and TG: HDL ratio, and lower HDL-C concentration, compared to those in the lowest one (*P* < 0.05). Also, individuals in the highest quartile of the EDIR score had higher intakes of refined grain, red and processed meat, vegetables, and fruits, but a lower intake of high-fat dairy. Dietary intakes of total energy and carbohydrates were significantly increased across EDIR score quartiles (*P* < 0.001), whereas intake of total fat was decreased (*P* < 0.001). There were no significant differences in any other demographic, anthropometric, biochemical, and nutritional measures across quartiles of the EDIR score.

**Table 2:** Baseline characteristics of participants according to quartiles (Q) of the empirical dietary index for insulin resistance

	Empirical dietary index for insulin resistance				*P* for trend*
	Q1(*n*=933)	Q2(*n*=933)	Q3(*n*=933)	Q4(*n*=933)	
**Demographic and Anthropometric data**					
Age (years)	42.6 ± 12.2	41.5 ± 12.3	40.1 ± 12.4	39.9 ± 11.9	<0.001
Male (%)	399 (42.8)	417 (44.7)	430 (46.1)	440 (47.2)	0.051
Physical activity (MET-h/week)	65.5 (31.9 – 94.3)	64.7(36.4 – 95.2)	71.1 (36.7 – 103.0)	71.4 (38.3 – 105.6)	<0.001
BMI (kg/m2)	27.0 ± 4.0	27.1 ± 4.0	27.1 ± 4.2	27.3 ± 4.2	0.165
waist circumference (cm)	91.8 ± 10.5	91.7 ± 11.0	92.0 ± 11.1	92.2 ± 11.2	0.401
Current smokers (%)	87 (9.3)	104 (11.1)	104 (11.1)	109 (11.7)	0.366
Academic education, N (%)	249 (26.9)	270(29.2)	304 (33.0)	266(28.8)	0.036
**Biochemical data**					
FBS(mg/dl)	92.9 ± 8.3	92.7 ± 8.4	92.5 ± 7.8	93.2 ± 8.7	0.393
TGs(mg/dl)	134 ± 82	134 ± 75	138 ± 80	145 ± 91	0.002
HDL- Cholesterol (mg/dl)	48.5 ± 11.5	47.8 ± 11.6	47.2 ± 11.5	46.9 ± 11.3	0.002
TG:HDL ratio	3.14 ± 3.01	3.16 ± 2.44	3.25 ± 2.40	3.50 ± 2.93	0.002
**Insulin Scores**					
EDIH	0.07 ± 0.22	0.13 ± 0.18	0.17 ± 0.19	0.23 ± 0.31	<0.001
ELIH	1.24 ± 0.23	1.27 ± 0.24	1.29 ± 0.25	1.32 ± 0.30	<0.001
EDIR	0.27 ± 0.13	0.50 ± 0.05	0.71 ± 0.07	1.20 ± 0.39	<0.001
ELIR	2.55 ± 0.58	3.25 ± 0.78	4.13 ± 1.06	6.07 ± 2.49	<0.001
**Nutrient Intake**					
Energy(Kcal/d)	2035 ± 660	2267 ± 660	2496 ± 652	2904 ± 703	<0.001
Carbohydrate(% of energy)	57.8 ± 7.3	59.0 ± 6.5	59.4 ± 12.8	59.7 ± 6.6	<0.001
Protein(% of energy)	15.0 ± 4.7	15.0 ± 3.0	15.3 ± 12.9	14.5 ± 2.5	0.138
Fat(% of energy)	31.0 ± 6.7	29.7 ± 5.8	30.5 ± 29.0	29.0 ± 6.3	0.020
**Food groups**					
Low-fat dairy (serving/d)	1.81 ± 1.34	1.80 ± 1.03	1.84 ± 1.11	1.89 ± 1.16	0.100
High-fat dairy (serving/d)	0.65 (0.21 – 1.33)	0.49 (0.16 – 1.04)	0.53 (0.18 – 1.03)	0.54 (0.20 – 1.10)	0.002
Refined grain(serving/d)	2.90 ± 1.46	3.76 ± 1.72	4.94 ± 2.33	7.36 ± 4.18	<0.001
Red and processed meat(serving/d)	0.56 ± 0.44	0.75 ± 0.56	0.90 ± 0.67	1.12 ± 0.99	<0.001
Fruits(serving/d)	3.28 ± 3.25	3.82 ± 3.62	3.87 ± 3.19	4.43 ± 3.77	<0.001
Vegetables(serving/d)	2.29 ± 1.69	2.99 ± 1.41	3.57 ± 1.78	4.74 ± 3.03	<0.001

Data represented as mean ±SD, or median (IQR 25-75) for continues variables and number and percent for categorical variables

*Chi-square and linear regression were used to test the trend of continues and categorical variables across quartiles of empirical dietary index for insulin resistance (as median value in each quartile), respectively

The association of EDIH, ELIH, EDIR, and ELIR with the risk of incident diabetes is reported in Table 3. In the age and sex-adjusted model, the odds of diabetes were higher in individuals of highest quartiles of the EDIR (OR = 1.62; 95 %CI:1.13–2.33, *P* for trend = 0.004), ELIH (OR = 3.10; 95 %CI:2.02–4.75, *P* for trend = < 0.001), and ELIR (OR = 1.78; 95 %CI:1.20 − 2.64, P for trend = 0.006) compared to those in the lowest quartile of these scores; However, there was no significant association between EDIH and risk of diabetes incident (OR = 93; 95 %CI:63–1.36, *P* for trend = 0.380). In model 2, after further adjustment for energy, WC, smoking, physical activity, education level, a higher score of EDIH showed no significant association with risk of diabetes; however, the positive associations of EDIR (OR = 1.72; 95 %CI:1.15–2.57, *P* for trend = 0.005), ELIH (OR = 3.07;95 %CI:2.00–4.76, *P* for trend = < 0.001), and ELIR (OR = 1.89; 95 %CI:1.25 − 2.88, *P* for trend = 0.006) with odds of diabetes remained significant. The associations remained significant after inclusion of biochemical variables in the model 3 and 4. After adjuting for all potential covariates, the odds of diabetes was 58 % higher for EDIR, 89 % higher for ELIH, and 74 % higher for ELIR in those in highest quartiles comrated to those in the lowest.

**Table 3:** The association between the insulin response dietary patterns and incidence of diabetes: the Tehran Lipid and Glucose Study

	Quartiles of scores				*P* for trend
	Q1	Q2	Q3	Q4	
**EDIH**					
Median score	-0.03	0.06	0.16	0.38	
Case/Total	69 / 933	80 / 933	55 / 933	53 / 933	
Model 1^a^	1.00 (Ref)	1.24 (0.88–1.75)	0.89 (0.61–1.29)	0.93 (0.63–136)	0.380
Model 2^b^	1.00 (Ref)	1.31 (0.93–1.86)	0.90 (0.62–1.32)	0.98 (0.66–1.45)	0.522
Model 3^d^	1.00 (Ref)	1.39 (0.96–2.01)	0.86 (0.58–1.29)	0.95 (0.63 1.44)	0.377
**EDIR**					
Median score	0.29	0.50	0.70	1.08	
Case/Total	58 / 933	58 / 933	64 / 933	77 / 933	
Model 1^a^	1.00 (Ref)	1.09 (0.74–1.59)	1.30 (0.89–1.89)	1.62 (1.13–2.33)	0.004
Model 2^b^	1.00 (Ref)	1.11 (0.75–1.64)	1.33 (0.90–1.97)	1.72 (1.15–2.57)	0.005
Model 3^d^	1.00 (Ref)	1.14 (0.76–1.72)	1.45 (0.96–2.19)	1.58 (1.03–2.44)	0.025
**ELIH**					
Median score	0.99	1.19	1.34	1.56	
Case/Total	31 / 933	55 / 934	70 / 934	101 / 933	
Model 1	1.00 (Ref)	1.76 (1.11–2.78)	2.03 (1.30–3.17)	3.10 (2.02–4.75)	< 0.001
Model 2^c^	1.00 (Ref)	1.76 (1.11–2.78)	2.02 (2.29–3.16)	3.07 (2.00–4.76)	< 0.001
Model 3^d^	1.00 (Ref)	1.35 (0.84–2.18)	1.30 (0.81–2.09)	1.89 (1.20–2.97)	0.004
**ELIR**					
Median score	1.96	3.12	3.29	3.61	
Case/Total	50 / 934	62 / 932	79 / 934	66 / 933	
Model 1^a^	1.00 (Ref)	1.46 (0.99–2.17)	2.21 (1.51–3.24)	1.78 (1.20–2.64)	0.006
Model 2^c^	1.00 (Ref)	1.49 (1.00–2.23)	2.32 (1.67–3.44)	1.89 (1.25–2.88)	0.006
Model 3^d^	1.00 (Ref)	1.44 (0.94–2.19)	2.19 (1.44–3.32)	1.74 (1.11–2.72)	0.031

^a^Model 1: adjusted for age and sex

^b^Model 2: additionally adjusted for energy, waist circumference, smoking, physical activity, and education level

^c^Model 2: additionally adjusted for energy, smoking, education level, and waist-adjusted BMI

^d^Model 3: additionally adjusted for for fasting blood sugar and TAG: HDL-cholesterol at baseline

## Discussion

Findings of the current study showed that higher scores of EDIR, ELIH, and ELIR were significantly associated with an increased risk of diabetes by 58 %, 89 %,, and 74 %, respectively after adjusting for potential confounding factors. However, no significant association was observed between EDIH and odds of diabetes. Based on our results, EDIR had a high correlation with ELIR (r:0.73), however EDIH had a moderate correlation coefficient with ELIH (r:0.40). Also the was a week but significant correlation coefficient between EDIR and EDIH (*r* = 0.30).

So it was to be expected that the ELIR and EDIR have similar effect size in predicting the risk of diabetes incident. Also, in general, associations for the lifestyle score were stronger than for the diet-only score, which fits with the explanation that in general, lifestyle is a stronger predictor of an insulin response than diet alone.

Studies on the association between hyperinsulinemic diet and developing diabetes in the worldwide limited to a cohort study conducted among US postmenopausal women [15]. Jin et al. reported that a higher score of EDIH was associated with a higher risk of diabetes incident. In contrast to their findings, we did not find a significant association between the score of EDIH and diabetes incident; although, the mentioned study had been conducted among elderly postmenopausal women. The non-significant findings of our study can be due to the low intake of EDIH components and consequently its low score estimation. Also, the different populations studied may be one of the causes of this consistency in results. We showed a considerable positive association between EDIR, ELIH, and ELIR and increased the risk of diabetes, which has not previously been studied. However, overall, some previous studies have reported a significant association between insulinemic potential of diet and lifestyle and elevated risk for other chronic diseases such as obesity and cancers [12–14]. Recently, a cohort study in American subjects has reported that high dietary insulinemic and inflammatory potential is associated with long-term weight gain or obesity in adults [14]. Also, the study of Tabung et al. [13] and Wang et al. [12] have revealed that higher insulinemic potential of diet or lifestyle is associated with increased risk of cancers such as digestive system cancers in both men and women.

Our findings are also compatible with the results of studies linking modifiable factors including adiposity, physical activity, and dietary pattern as components of the insulinemic potential of diet and lifestyle score with hyperinsulinemia, and risk of diabetes. The Weyer et al. study indicated that adiposity is associated with the degree of hypo-adiponectinemia, IR, and hyperinsulinemia and can increase the development of diabetes [20]. Also, it has been reported that physical inactivity (and unhealthy nutrition) is potentially associated with increased hyperinsulinemia and then becomes the leading cause of diabetes via distorting body composition and weight gain [21]. Studies have shown that dietary patterns with high insulin secretion ability such as the western diet in individuals with an unhealthy lifestyle could due to the developing incidence of diabetes and other chronic diseases [22, 23]. It also appears to be a low insulinemic dietary pattern characterized with higher intake of whole fruits, vegetables, and leafy green vegetables (rich in fiber, calcium, magnesium, potassium), and lower intakes of red meat, processed meats, sugar-sweetened beverages, refined grains, and chicken is associated with decreased hyperinsulinemia [11, 14].

In current study, we have adjusted the analysis of data for baseline values of FBS and TGs:HDL-C. Because the high baseline values ​​of these variables in some individuals, even if they do not have diabetes and dyslipidemia, could be effective in increasing the risk of diabetes in them as a high-risk group. After controlling the confounding effect of baseline level of FBS and TGs:HDL-C, although the main results were slightly attenuated, they still remained significant. This decrease in odds of diabetes was mostly observed acording to qaurtiles of ELIH and ELIR; because, ORs values for ELIH and ELIR were larger than the ORs values of EDIH and EDIR, so a greater change was observed in the ORs of these insulinemic potential of lifestyle indices. Also, insulinemic potential of lifestyle (assessed by ELIH and ELIR) are determined based on non-nutritional factors such as BMI and adiposity, which is expected that the baseline values of FBS and TGs:HDL-C will be more effective on these determinants of ELIH and ELIR.

C-peptide is commonly preferred to insulin measurement for assessing β-cell function. C-peptide is produced in equal amounts to insulin and can be used to assess endogenous insulin secretion; thus it is considered as a valid marker of hyperinsulinemia in the long-term period. Also, in patients treated with insulin, for the distinction between exogenous and endogenous insulin, C-peptide measurement must be used. Insulin produced by the pancreas is extensively (approximately 50 %) first-pass metabolized by the liver, both the extent of the first-pass metabolism and peripheral clearance of insulin is variable, therefore peripheral insulin levels may not accurately reflect portal insulin secretion. Even in non-insulin-treated patients, peripheral C-peptide levels more accurately reflect portal insulin secretion than measurement of peripheral insulin [24]. Therefore, it can be said that in the current study we used strong and valid indices including ELIH and EDIH, which were calculated and validated based on C-peptide as a valid and less available marker. Determination and validation of these indices with C-peptide reduces the chance of error in assessing the association between the insulinemic potential of diet and lifestyle with diabetes incident. However, Considering that data on C-peptide are less available in cohort studies and are more expensive, Therefore, we could not assess the correlation between EDIH and ELIH due to lack of C-peptide measurements and this can be considered a weakness for our study.

It should be noted that, despite the above mentioned limitation on C-peptide measurements, in our study the direction and strength of association between insulinemic potentials of diet and lifestyle indices and risk of diabetes are similar to the other two previous studies among NHS and HPFS concerning digestive system cancers. It shows an acceptable agreement between insulinemic indices between our study and studies among NHS and HPFS cohorts. Furthermore, EDIR and ELIR have been validated using TG:HDL in the data of HPFS and NHS-II studies. For validation study, authors have used the NHS data and observed that similar to participants in the HPFS and NHS-II, the concentrations of TAG:HDL-cholesterol increased monotonically across quintiles of EDIR and ELIR in participant characteristics of the NHS [11]. Based on findings in Table 2, our study also has shown that tha ratio of TGs:HDL-C is increased across quartiles of EDIR. This result could be interpreted as a simple validity of EDIR in the present study. Finally, considering that, some previously presented indices such as empirical dietary inflammatory pattern (EDIP), which firstly validated among US population, after using in other populations and confirmed its performance for predicting the inflammatory potential of diet, recently its global validation published [25]. These insulinemic potentials of diet and lifestyle also should be tested in other populations to assess those performances.

Although the mechanisms underlying the role of the insulinemic potential of diet and lifestyle indices on the risk of diabetes are not yet fully understood; however, the insulinemic effect of food components intakes in combination with BMI status and physical inactivity as a major part of an individual’s lifestyle can play a crucial role in long-term insulin secretion. It has been demonstrated that high chronic insulin secretion due to high consumption of insulinogenic foods, high level of adiposity, and sedentary life during a long period as through the development of fat mass and IR can lead to disruption of pancreatic beta cells and consequently incidence of diabetes [14, 26]. Moreover, high insulin concentrations can suppress lipolysis and stimulate glucose uptake that could lead to developing diabetes [27].

Several strengths of our study should be noted. To the best of our knowledge, the current study is the first population-based cohort study that assessed the association between the empirical food-based dietary indexes for hyperinsulinemia and lifestyle score (EDIH, ELIH, EDIR, and ELIR) and the incidence of diabetes in the Middle East and North Africa region. Also, we defined diabetes by objective measurements, including FBS and 2-h post 75 g glucose load, and it was not based on self-reporting. Other strengths of this study were the appropriate duration of follow-up of our study and using a valid and reliable FFQ and physical activity questionnaire. Despite these strengths, this study is not without limitations. Although we used a valid and reliable FFQ to estimate nutritional intakes, the probability of a measurement error is unavoidable. Furthermore, despite adjusting for the confounding variables in our study, residual confounding due to unknown or unmeasured confounders cannot be excluded.

## Conclusions

Our findings indicated that higher scores of ELIH, ELIR, and EDIR are associated with an increased risk of diabetes. However, there is no significant association between EDIH and the risk of developing diabetes. Overall, our findings support that the insulinemic potential of diet and lifestyle may influence the incidence of diabetes in adults through mechanisms involving insulin signaling pathways. Therefore, nutritional and lifestyle modifications aimed at reducing the insulin concentrations or insulin resistance can reduce the risk of developing diabetes.

## Data Availability

The datasets analyzed in the current study are available from the corresponding author on reasonable request.

## Abbreviations

ADA: American Diabetes Association
BMI: Body mass index
CI: Confidence interval
EDIH: Empirical dietary index for hyperinsulinemia
EDIR: Empirical dietary index for insulin resistance
ELIH: Empirical lifestyle index for hyperinsulinemia
ELIR: Empirical lifestyle index for insulin resistance
FBS: Fasting blood sugar
FCT: Food composition table
FFQ: Food frequency questionnaire
HDL-C: Serum high-density lipoprotein-cholesterol
IQR: Interquartile range
IR: Insulin resistance
OR: Odds ratio
TLGS: Tehran Lipid and Glucose Study
TGs: Triglyceride
TC: Total cholesterol
USDA: Using the United States Department of Agriculture
WC: Waist circumference
